# Clinical management of patients diagnosed with acute myeloid leukemia treated with venetoclax in combination with hypomethylating agents after achieving a response: a real-life study

**DOI:** 10.1007/s00277-024-05923-5

**Published:** 2024-08-29

**Authors:** Carlos Jiménez-Vicente, Ares Guardia-Torrelles, Amanda Isabel Pérez-Valencia, Alexandra Martínez-Roca, Sandra Castaño-Diez, Francesca Guijarro, Albert Cortés-Bullich, Beatriz Merchán, Ana Triguero, Isabel Hernández, Helena Brillembourg, Daniel Munárriz, Inés Zugasti, Francesc Fernández-Avilés, Marina Diaz-Beyá, Jordi Esteve

**Affiliations:** 1 Hematology Department, Hospital Clínic de Barcelona, Villarroel 170, 08036 Barcelona, Spain; 2 Institut d’Investigacions Biomèdiques August Pi I Sunyer (IDIBAPS), Barcelona, Spain; 3 Pathology Department. Hospital Clinic, Hemopathology Unit, Barcelona, Spain; 4 University of Barcelona, Barcelona, Spain

**Keywords:** AML, Venetoclax, Management, Cytopenias, Adverseevents

## Abstract

Although there is an approved indication for venetoclax and hypomethylating agents (VenHMA) and its use in different AML settings will be expanded in the following years, the management of the adverse events (AEs) lacks of harmonized algorithms during treatment of these patients. We have studied the incidence of relevant AEs of 43 patients who achieved a response to VenHMA and its management. Median overall survival of our cohort was 19 months. No patients discontinued treatment due to AEs after C3D1, Regarding severe AEs, high rates of grade 4 neutropenia (97.6%) and grade 4 thrombocytopenia (65.1%) were observed. Severe infectious AEs rate was 16%. Due to severe myelotoxicity, most patients required a progressive dose reduction of both venetoclax and hypomethylating agents during follow-up, being 87.8% at C6D1. Transfusional dependence rate was 91% and G-CSF was prescribed to 86% of the patients. Finally, there was not a significant difference in hemoglobin, platelets and absolute neutrophil count after achieving complete response comparing paired samples during follow-up, although cytopenia rate was high during initial follow-up. We conclude that dose reduction of VenHMA after achieving a response in patients diagnosed with AML is required in most patients and essential to avoid prolonged cytopenia-related adverse events and a rapid and standardized method on how to perform it might decrease the AEs rate.

## Introduction

Since the publication of the phase III Viale-A trial in 2020 [1], the combination of the BCL-2 selective inhibitor venetoclax and azacytidine (VenAza) has become the gold standard frontline treatment for patients diagnosed with acute myeloid leukemia (AML) ineligible for intensive chemotherapy, also known as unfit patients. In the trial, VenAza proved to have an impressive remarkable antileukemic effect, with better response rate (66% vs. 28%, p < 0.001) and an increased median overall survival compared to azacitidine in monotherapy (14.7 vs. 9.6 months, p < 0.001), along with a feasible safety profile.

In the safety analysis of the Viale-A study, 83% of patients in the VenAza group experienced at least one grade 3 or higher adverse event (AE). Hematologic AEs such as thrombocytopenia (in 46% of the VenAza patients), neutropenia (42%), febrile neutropenia (42%) and anemia (26%), were more frequently reported in the VenAza group compared to the control one. Dose interruptions were prescribed in 53% of the patients in the VenAza group due to febrile neutropenia or cytopenias during the follow-up of the study. Interestingly, only 1% of the patients included in the VenAza group developed tumor lysis syndrome (TLS), which was the most relevant AE observed when venetoclax was prescribed for other hematological malignancies [2]. Despite these AEs, the mortality rate at 30 days was low (7%), showing the feasibility of the combination in this setting. Several real-life cohorts, including the series of patients treated in our center [3, 4], have shown a similar safety profile afterwards, highlighting the relevance of proper management of the AEs to avoid discontinuation, especially during the first cycles of treatment until a disease response is obtained.

In addition to the combination with azacitidine, other combinations with low-intensity chemotherapy agents have shown a strong antileukemic effect, such as the combination with decitabine or with low-dose cytarabine [5–8], and recently, after the approval of VenAza in the unfit setting, several studies have also shown optimistic response rates and a feasible low toxicity profile in different AML settings, expanding the therapeutical spectrum of the venetoclax-based combinations in this disease. Some examples of this broader use are the use of VenAza in patients diagnosed with R/R or AML evolving from MPN [9], in patients diagnosed with higher risk MDS, in triplets including FLT3i, IDHi or menin-inhibitors [10–12] *FLT3 *[13, 14]), in combination with standard chemotherapy in different subsets of patients diagnosed with AML [15–17], or as maintenance strategies) [18–21].

Although there is an approved indication for VenAza and its use in different AML settings will be expanded in the following years, the management of the AEs lack of harmonized algorithms during treatment of these patients, with a discordant approach among centers in relevant topics such as the use and type of antimicrobial prophylaxis. Also, the long-term follow-up AEs of low-intensity venetoclax-based combinations after the initial response have not been deeply studied yet in the real-life setting and the relevant data we have obtained nowadays is based on the long-term follow up of the Viale-A [22].

In this study, we have studied the incidence of relevant AEs after induction of patients diagnosed with AML treated with venetoclax in combination with hypomethylating agents (VenHMA) and its management, focusing mainly on treatment dose reduction through follow-up in order to avoid the discontinuation until progressive disease.

## Patients and methods

We performed a retrospective, single-center study with patients, older than 18 years, treated with VenHMA at the Hospital Clínic of Barcelona between March 2019 and October 2023. Patients received standard dose of venetoclax (400 mg PO daily for 14–28 days and 200 mg in those patients who received moderate CYP3A inhibitors isavuconazole or amiodarone (n = 5) during the first two cycles of treatment) and azacytidine (75 mg/m^2^ SC, 7 days each cycle of 28 days) or decitabine (20 mg/m^2^ EV, 5 days), either for newly-diagnosed AML (ND-AML) or refractory/relapsed AML (R/R AML).

Patients enrolled in clinical trials were excluded from the analysis. All baseline characteristics and clinical course during treatment were retrospectively collected from the electronic records in our center. Cutoff data to analyze follow-up was the 21st March 2024.

Our primary endpoint was to analyze the dose reduction rate in patients treated with VenHMA who achieved a morphological complete response and received at least four complete cycles of treatment. Secondary objectives included the analysis of adverse events rate during follow-up after the initial two cycles of treatment and the impact of the dose reduction in the adverse events rate. The study was approved by the Ethics Committee of the Hospital Clinic of Barcelona and was in accordance with the Declaration of Helsinki.

AML was classified according to the ICC 2022 classification of myeloid neoplasms and the WHO 5th classification of myeloid neoplasms [23, 24]. AML disease risk stratification and response criteria during treatment were assessed according to the 2022 European LeukemiaNet risk criteria [25] (ELN 2022). The disease was reassessed at bone marrow at least once of each three cycles of treatment, following the latest ELN 2022 guidelines based on the Cheson criteria [25, 26]. According to these criteria, the overall response term used in this study included complete response (CR), complete response without a hematological recovery (CRi), morphological leukemia free status (MLFS) and partial response (PR). Measurable residual disease (MRD) was analyzed according to the 2021 ELN criteria [27]. Performance status (PS) was assessed according to the Eastern Cooperative Oncology Group (ECOG) score [28].

### Treatment dosage and adjustment

Initial treatment was adjusted for patients taking CYP3A4 inhibitors. Initial ramp-up of venetoclax (3 days of progressive increasing dose up to maximal dose) together with adequate tumor lysis syndrome (TLS) prophylaxis during the first days of cycle 1. Before C3D1, antimicrobial prophylaxis with quinolone and triazoles was administered to all patients according to our institutional protocols and suspended if there was a complete recovery of absolute neutrophil count after cycle 2 (> 1 × 10^9/L). Antimicrobial prophylaxis due to grade 4 neutropenia during follow-up was prescribed at the discretion of the clinicians.

AEs were recorded from the moment of initial treatment and evaluated for this study from the first day of the third cycle of treatment (C3D1). Adverse events were determined according to the Common Terminology Criteria for Adverse Events (CTCAE), Version 4.03, May 2009.

Dose reduction during follow-up was performed if any of the following events was observed during the previous cycle: grade 4 neutropenia (< 0.5 × 10^9^/L) which needed of G-CSF administration to shorten its duration, transfusion-dependent thrombocytopenia or anemia or any other grade 2 to 4 adverse events likely related to VenAza dosage. Dose reduction was standardized from 2020 onwards with the development of a hospital specific protocol, using a three steps following scheme, diminishing first the venetoclax number of days, afterwards the hypomethylating agent (HMA) dose and finally a new reduction in the venetoclax dose. Furthermore, additional reduction in both venetoclax or HMA if cytopenias persisted and the complete response was still maintained before the next cycle. Cycles were postponed until neutrophil blood count recovery (> 1 × 10^9^/L) and a platelet count over 50 × 10^9^/L.

### Statistical analysis

Dose reduction, response rate and adverse events were measured after cycles 2, 4 and 6. Descriptive statistical analysis on all patients was performed. Median and range were used for continuous variables and frequency and percentage for categorical variables. Fisher’s exact test or χ^2^ test were used for univariate analysis in categorical variables and paired samples Wilcoxon rank sum test was used for to compare continuous variables at different times during follow-up. All p values were two-sided with statistical significance evaluated at the 0.05 alpha level. All statistical analyses were performed with R statistics version 4.0.3 (R core Team, R Foundation for Statistical Computing, Vienna, Austria).

## Results

### Baseline characteristics and disease response

A total of 124 patients diagnosed with AML or MDS/AML have been treated with venetoclax in combination with hypomethylating agents in our center between March 2019 and October 2023. Ten patients were included in clinical trials and, therefore, excluded of our study. Furthermore, fifteen patients were treated due to positive MRD and also excluded from the study, and three more patients were treated with VenHMA as consolidation of a previous standard chemotherapy induction, due to prolonged cytopenias. Finally, 96 patients fulfilled inclusion criteria. The overall response rate (ORR) was 45.8% (44/96), with a 70% in ND-AML (28/40), and 28.6% in R/R AML (16/56).

**Fig. 1:**
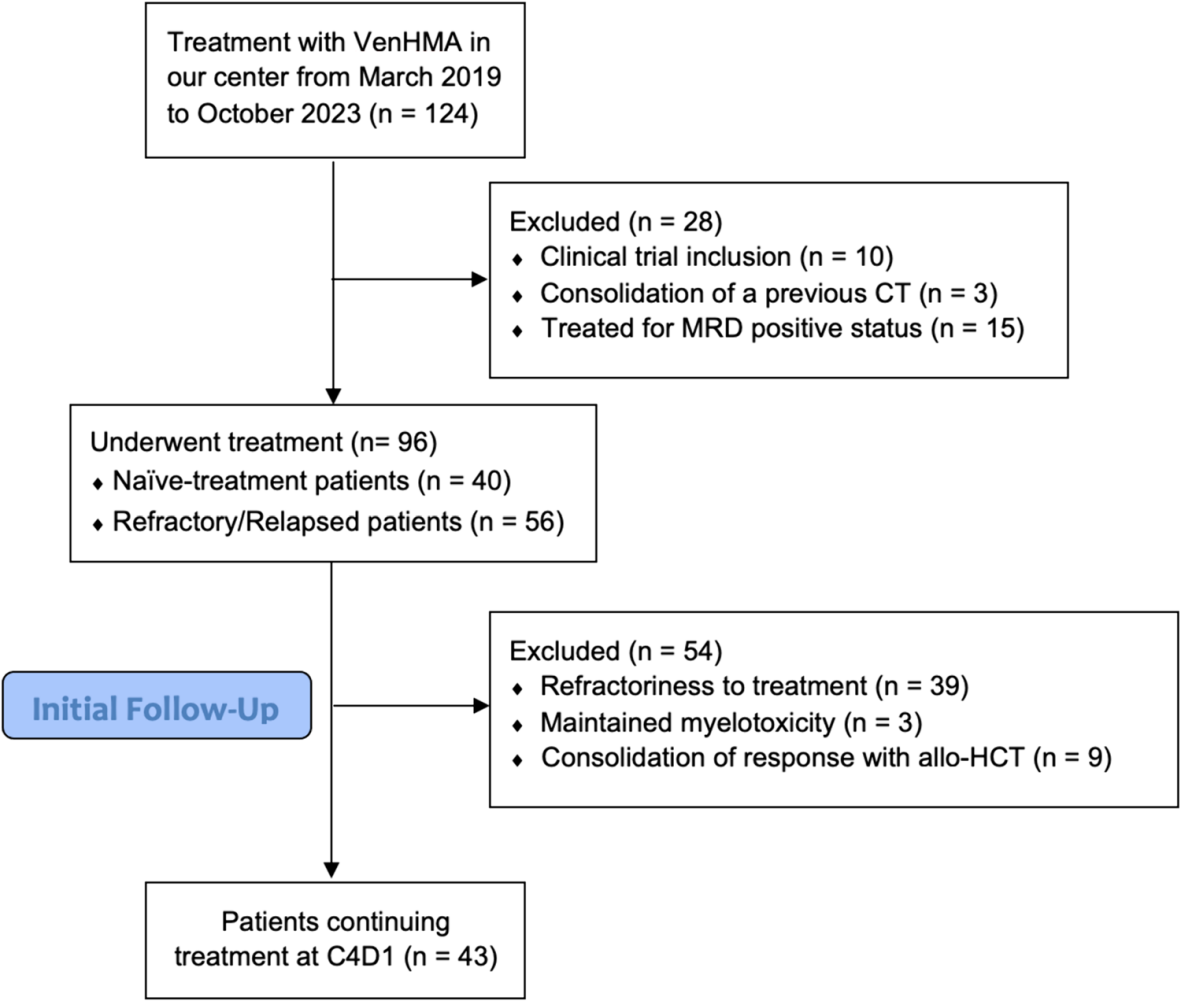
Flow chart of all patients treated with venetoclax in combination with hypomethylating agents in the Hospital Clinic of Barcelona since March 2019

Fifty-three patients (55% of the initially included patients) discontinued treatment before finishing cycle 4. Reasons to discontinue treatment were: refractoriness to treatment in the 74% (39/53) of the patients after completing one or two cycles, maintained grade 4 myelotoxicity in 5 out of the 53 patients (9%) (including 2 patients with COVID-19 infection during the first wave in Spain in March 2020 and one patient presenting with a subdural hematoma during aplasia) and consolidation of the achieved response with an allogeneic hematopoietic stem cell transplantation (allo-HCT) (17%, 9 patients). Finally, 43 patients were included in the study (Fig. 1).

Baseline characteristics and response to treatment of all patients are displayed on Table 1. Briefly, 60.5% were males with a median age of 73 years (range 39–84 years). The most prevalent subgroups were patients diagnosed AML with myelodysplasia-related gene mutations (44.1%), AML, NOS (18.6%) and AML with mutated *TP53* (16.3%),

**Table 1:** Baseline characteristics of all patients. ICC: International Consensus Classification of Myeloid Neoplasms. ELN: European LeukemiaNet, ECOG PS: Eastern Conference Oncology Group performance status, C1D1. First day of first cycle of treatment C4D1: First day of forth cycle of treatment, ANC: Absolute Neutrophil Count

	**Patients (N = 43) (%)**
**Age, median (range)**	73 (47–83)
**Sex, n (%)**	
Male	18 (41.9)
Female	25 (58.1)
**Diagnosis (ICC 2022), n (%)**	
AML or MDS/AML with myelodysplasia related gene mutations	19 (44.1)
AML or MDS/AML, NOS	8 (18.6)
AML or MDS/AML with mutated *TP53*	7 (16.3)
AML with mutated *NPM1*	6 (14)
AML with myelodysplasia-related cytogenetical abnormalities	1 (2.3)
AML with in-frame BZip *CEBPA* mutations	1 (2.3)
AML with *inv(16)*	1 (2.3)
**Risk genetic scale (ELN2022), n (%)**	
Favorable	7 (16.3)
Intermediate	7 (16.3)
Adverse	29 (67.4)
**ECOG PS 0–1 C1D1, n (%)**	25 (58.1)
**ECOG PS 0–1 C3D1, n (%)**	40 (93)
**Disease status**	
Frontline therapy	32 (74.4)
Refractory/Relapsed	11 (25.6)
**Previous treatment received, n (%)**	
Frontline therapy	32 (74.4)
Intensive chemotherapy (IC)	4 (9.3)
IC + Allogeneic stem cell transplantation	1 (2.3)
Hypomethylating agent-based regimens	9 (20.9)
**Number of previous treatments received, median (range)**	0 (0–2)
**Hypomethylating agent, n (%)**	
Azacitidine	28 (65.1)
Decitabine	15 (34.8)
**Overall response rate, n (%)**	43 (100)
Complete response with negative MRD	18 (41.9)
Complete response with positive MRD	9 (20.9)
Complete response without hematologic recovery	14 (32.6)
Partial response	2 (4.6)
**Hematology at C3D1, median (range)**	
ANC (× 10^9^/L)	
Hemoglobin (g/dL)	
Platelets (× 10^9^/L)	

Among the 43 patients, 32 (74.4%) received VenHMA as ND-AML, while the remaining 11 (25.6%) were R/R AML patients. According to the ELN2022, 67.4% of the patients were included in the adverse risk group with 16.3% having an intermediate risk, and another 16.3% a favourable risk. At baseline, only 58.1% of the patients (25/43) had an ECOG PS score of 0 or 1, while the percentage of patients with a ECOG PS score lower than 2 at C3D1 was 93% (40/43). Azacitidine was the most common hypomethylating agent, being used in the 65.1% of the patients (28/43).

**Fig. 2:**
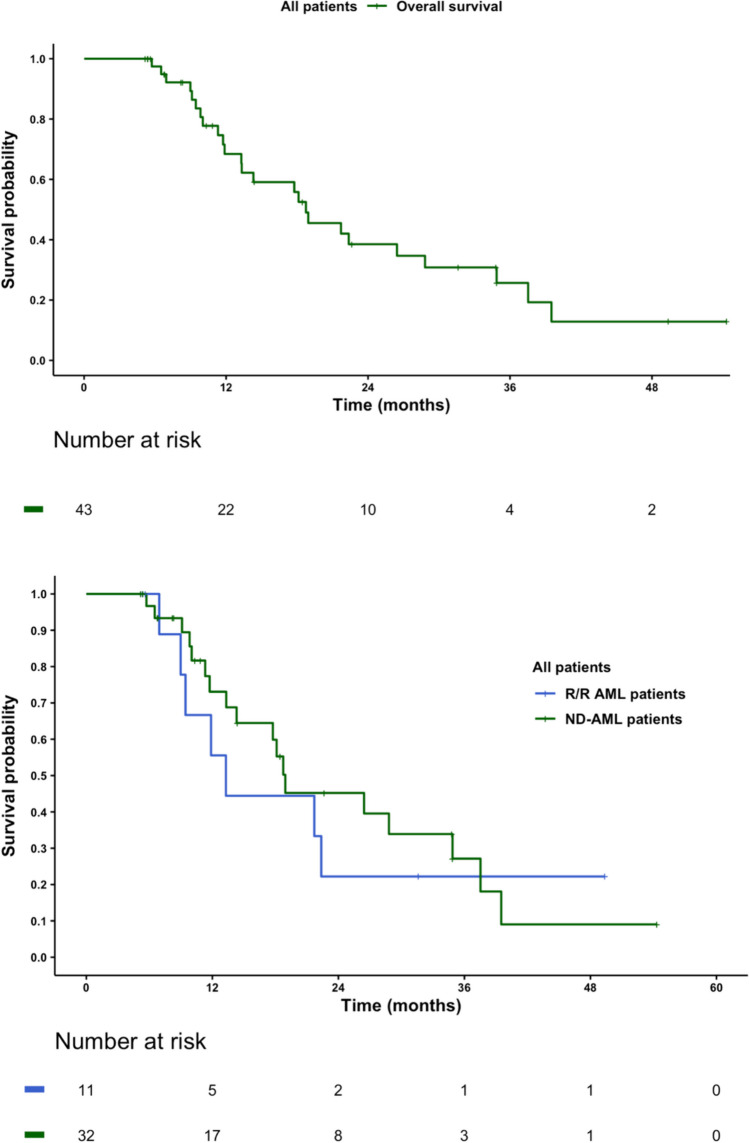
Overall survival of all patients in the study and overall survival depending on treatment status

According to the response, 18 patients (41.9%) achieved a CR with negative measurable residual disease, 9 patients (20.9) a CR with persistent positive MRD, 14 patients (32.6) had a CRi and finally, 2 patients (4.6) achieved a PR. Median number of cycles to achieve any response was 1 (range 1–3). The median number of prescribed VenHMA cycles during follow-up was 6 (range 4–34), with a median follow-up from initial treatment of 13.3 months (range 5–54). Median overall survival of the whole cohort was 19 months (95% CI: 13.3–37.5) with no statistical difference between patients who had received a previous treatment (p = 0.85) (Fig. 2A and B).

### Adverse events during treatment

No patients discontinued treatment due to AEs after C3D1. Regarding AEs during follow-up after C3D1, myelotoxicity was the most frequent. with 97.6% (42/43) and 65.1% (28/43) of the patients having grade 4 neutropenia and grade 4 thrombocytopenia respectively. During follow-up, due to maintained expected neutropenia, G-CSF was prescribed to 86% of patients, with a median usage of 5 cycles (range 1–16).

While the grade 4 neutropenia rate was 97.6%, the grade 3–4 infection rate after C3 was 16% (6/43). Osteomyelitis, skin infection, febrile neutropenia in two patients and COVID-19 in three patients were the infections observed in our cohort. All of these patients required a hospital admission. There were no other reasons for hospital admission after C3D1 in our cohort. Interestingly, one of the six patients stopped treatment due to repeated infectious events after cycle 4 and in other case, there were 3 repeated infections during last cycle of treatment after a prolonged response, before a progressive disease was observed at the next bone marrow assessment.

Cardiovascular AEs were only observed in one case, who presented with atrial fibrillation during follow-up, without an interruption of the treatment. All adverse events are displayed in Table 2.

**Table 2:** Adverse Events during follow-up in all patients treated with venetoclax and hypomethylating agents for at least 4 cycles of treatment

Adverse events after C3D1	All patients (N = 43)
**Neutropenia, n (%)**	42 (97.6)
Grade 3	1 (2.3)
Grade 4	41 (95.3)
Patients who have required administration of G-CSF	42 (97.6)
Treatment cycles requiring G-CSF administration, median (range)	4 (1–12)
**Anemia, n (%)**	25 (58.1)
Grade 3	24 (55.8)
Grade 4	1 (2.3)
**Thrombocytopenia, n (%)**	35 (81.4)
Grade 3	7 (16.3)
Grade 4	28 (65.1)
**Grade 3–4 infectious diseases, n (%)**	6 (14)
Osteomielitis	1 (2.3)
Celulitis	1 (2.3)
Febrile neutropenia	2 (4.6)
COVID-19	3 (6.9)
**Atrial fibrillation, n (%)**	1 (2.3)

### Dose reduction rate and cytopenia follow-up during treatment

During the first two cycles of treatment, 25.6% of the patients (11/43) had already been prescribed a dose reduction of treatment. At this moment, only venetoclax was reduced in all of them.

After 3 cycles of treatment a dose reduction was prescribed in 18 patients (41.9%), with a dose reduction of both treatments in two patients, and the remaining 16 patients having only a dose reduction of venetoclax.

After cycle 4, the dose reduction rate was 48.8% (21/43), with three patients presenting a reduction in both venetoclax and HMA. At this moment, 7 patients discontinued treatment, 4 due to consolidation of the response undergoing allo-HCT, 2 due to progressive disease, and the remaining one due to repeated infectious events during despite a morphological response achieved. The remaining 4 patients were undergoing cycle 5 at cutoff data. Venetoclax (at a standard dose of 400 mg) during 14 days and azacitidine 75 mg/m^2^ during 7 days of 28-day cycles was the most prescribed dose scheme at this moment.

**Fig. 3:**
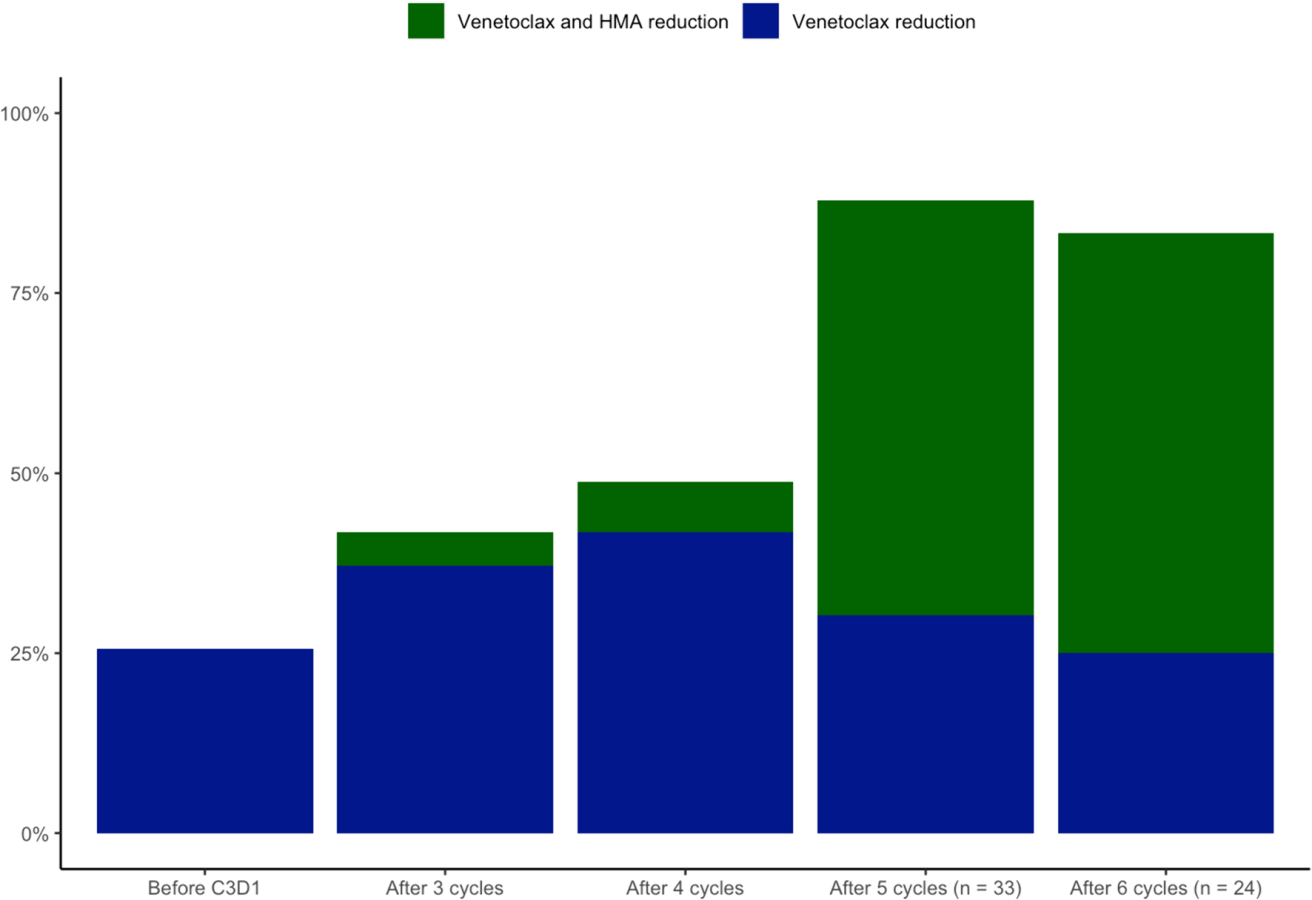
Dose reduction rate (percentage) of venetoclax and/or hypomethylating agent (HMA) in treated patients during the follow up, after two, three, four, five and six cycles of treatment (see legend)

After cycle 5, 87.8% of the remaining patients on treatment required at least one dose reduction step of treatment, with more patients having a reduction in both venetoclax and HMA than only in the BCL-2 inhibitor (57.8% vs. 30.3%). Interestingly, there were only 4 patients who were still on the initial dose, who were the same patients without a dose reduction after cycle 6, which explains the lower relative percentage of dose reduction after cycle 6 than after cycle 5, since there were patients who stopped treatment due to progressive disease or allo-HCT after this cycle. After cycle 6, venetoclax 200 mg during 14 days and azacitidine 75 mg/m^2^ during five days in 28-day cycles was the most prescribed dose. The median number of reduction steps/patient during follow-up was 2 (range 1–4). Dose reduction after cycles is displayed in Table 3 and Fig. 3.

**Table 3:** Dose adjustment during cycles and transfusional independence during follow-up in all patients treated with venetoclax and hypomethylating agents for at least 4 cycles of treatment

	**Number (%)**
Dose adjustment	
After 2 cycles of treatment (n = 43)	11 (25.6)
Reduction of Venetoclax	11 (25.6)
Reduction of Venetoclax and Hypomethylating agents	0 (0)
After 3 cycles of treatment (n = 43)	18 (41.9)
Reduction of Venetoclax	16 (37.2)
Reduction of Venetoclax and Hypomethylating agents	2 (4.6)
After 4 cycles of treatment (n = 43)	21 (48.8)
Reduction of Venetoclax	18 (41.9)
Reduction of Venetoclax and Hypomethylating agents	3 (6.9)
After 5 cycles of treatment (n = 33)	29 (87.8)
Reduction of Venetoclax	10 (30.3)
Reduction of Venetoclax and Hypomethylating agents	19 (57.8)
After 6 cycles of treatment (n = 24)	20 (83.3)
Reduction of Venetoclax	6 (25)
Reduction of Venetoclax and Hypomethylating agents	14 (58.3)
Reduction steps/patient during follow-up, median (range)	2 (1–4)
Transfusional Independence after 6 or more cycles (n = 24)	22 (91.7)

The transfusion independence rate after 6 cycles was 91.7%. Finally, though the high rate of adverse events related to myelosuppression, there was not a significant decrease in the mean hemoglobin level (Hb) absolute neutrophil count (ANC) and platelet count (Plat) once a response was achieved comparing C3D1 and C5D1 (Hb: 109 g/L (74–139) vs. 110 g/L (89–145), p = 0.12, ANC: 1.9 (0.1–21.7) vs. 1.64 × 10^9^/L (0.2–17), p = 0.76 and Plat: 163 × 10^9^/L (32–548) vs. 120 × 10^9^/L (26–452), p = 0.005), while peripheral blood values were increased from baseline data (C1D1 vs. C5D1, Hb: 92 g/L (71–143) vs. 110 g/L (89–145), p < 0.001, ANC 0.8 × 10^9^/L (0–12) vs. 1.64 × 10^9^/L (0.2–17), p < 0.001 and Plat: 52 × 10^9^/L (7–406) vs. 120 × 10^9^/L (26–452), p = 0.005).

## Discussion

**Fig. 4:**
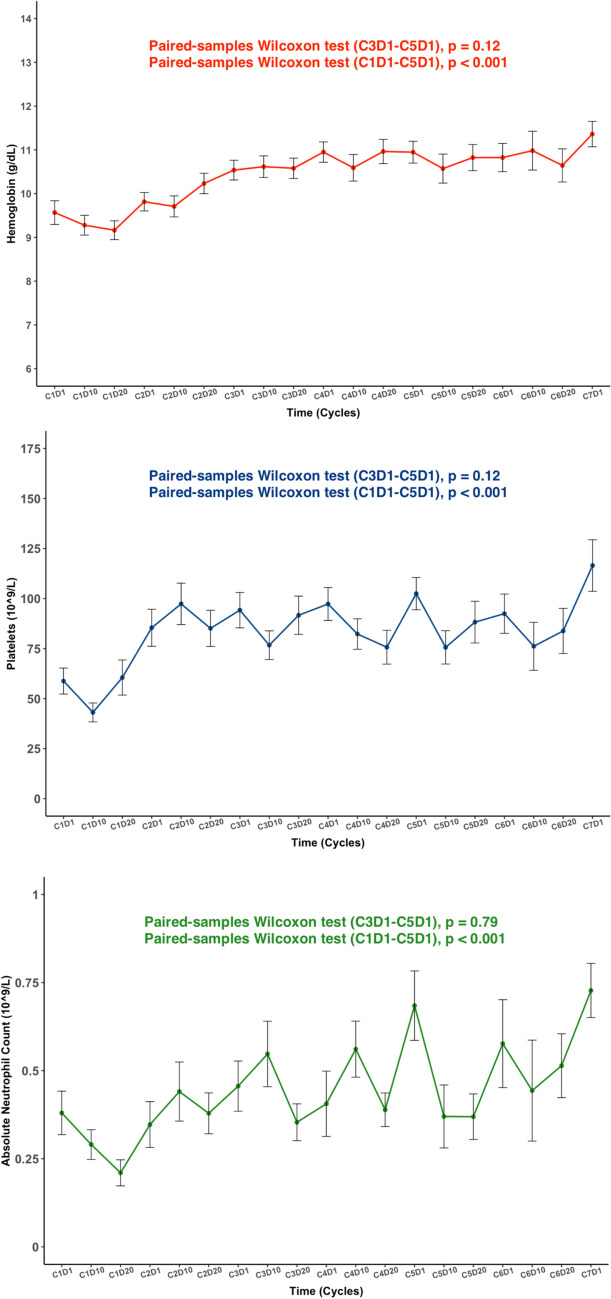
Mean (± standard error) of hemoglobin (g/dL), absolute neutrophil count (× 10^9^/L) and platelet count (× 10^9^/L) during follow-up. Paired samples Wilcoxon test was used to analyze the difference during treatment

VenAza has become the gold standard in unfit patients diagnosed with AML. and is also being explored in different scenarios, meaning a higher burden of patients to treat. Though an eventual relapse is expected in the vast majority of patients based on the long-term follow-up of the phase Ib and Viale-A studies [29, 30], a response lasting for months is expected in most of these patients [31], being especially prolonged in some subgroups of patients such as those with mutated *IDH2* and/or *NPM1* [32–34]. Prolonged myelosuppression has been repeatedly reported in clinical trials [1, 5, 8, 35] and real-life series [36–41] as the most frequent adverse event during treatment, and thus, prophylactic measures such as the use of azoles to prevent fungal infection, which requires venetoclax dose adjustment due to CYP3A cytochrome interaction [42–44] or blood transfusions have been adopted in most of the treating institutions (Fig. 4).

Once a response is achieved, venetoclax dose is decreased and the interval between cycles is extended in most treated patients to allow hematological recovery. Nonetheless, standardization of dose reduction and delay of cycle initiation due to cytopenia during follow-up is still lacking.

The high grade 4 neutropenia and thrombocytopenia rate (83.1% and 53.5% respectively) observed in the series have leaded to a rapid dose decrease after achieving a morphologic complete response in order to avoid myelosuppression, with an anticipated approach compared to the recommended in the European Public Assessment Report of venetoclax. Thus, reduction was instituted in this population after the first episode of prolonged cytopenia, instead of keeping on with the same dose after a first episode of prolonged cytopenia. To highlight, sixty percent of the patients required at least one dose decrease after one cycle and almost 90% of all patients needed a dose reduction after 6 cycles. Two thirds reduced both venetoclax and hypomethylating agents, with a median number of 2 reduction steps per patient, and G-CSF was widely used.

Although neutropenia and thrombocytopenia rates were higher than in most of the published data [1, 5, 35–37, 39–41, 45, 46] grade 3 or 4 infectious diseases rate after two cycles was relatively low (16%). The myelotoxicity caused by the standard initial dose of venetoclax-based low-intensity treatments in combination with the persistence of the disease seem to be the higher risk factors to present adverse events related to VenHMA, since the overall infection rate during all treatment including the first two cycles in this series of patients is similar to the previous publications[3, 4]. It is important to remind that the main goal of the treatment is to achieve a response and, therefore, the initial dose must be maintained until the response is assessed, but, once this milestone is confirmed, a dose reduction can be safely established in case of persistent cytopenia to avoid myelosuppression-derived complications. This dose reduction will eventually help not only to avoid adverse events but also to improve the quality of life of the patients, since transfusions and concomitant treatment such as G-CSF or antimicrobial prophylaxis may not be needed anymore during the response. The benefit of this pro-active dose reduction policy is exemplified in the maintained peripheral blood values after the initial response without any myelosuppressive effect added and the significant rise at C5D1 compared to baseline level, as well as the high transfusion independence rate.

The retrospective nature of the study and the limited size of the study cohort are limitations of the present study. Further knowledge with prospective trials comparing different treatment dosage strategies during follow-up and adverse events rate are needed to clarify which is the adequate dose decrease in patients to balance a maintained response with the lowest adverse events rate possible.

Finally, we conclude that dose reduction of VenHMA after achieving a response in patients diagnosed with AML is required in most patients and essential to avoid prolonged cytopenia-related adverse events. Further knowledge with prospective data is needed to obtain stronger evidence.

## Data Availability

Due to privacy and ethical concerns, the data that support the findings of this study are available upon request from the corresponding author.

## References

[1] DiNardo CD et al (2020) Azacitidine and venetoclax in previously untreated acute myeloid leukemia. N Engl J Med 383:617–629 32786187 10.1056/NEJMoa2012971

[2] Seymour J et al (2018) Venetoclax-rituximab in relapsed or refractory chronic lymphocytic leukemia. N Engl J Med 378:1107–1120 29562156 10.1056/NEJMoa1713976

[3] Jimenez-Vicente C et al (2021) Real-life preliminary experience of treatment of newly-diagnosed and refractory/relapsed (R/R) acute myeloid leukemia patients with combination of venetoclax with hypomethylating agents. Hemasphere 5(S2):197–198

[4] Brillembourg H et al (2022) Infecciones durante el tratamiento de venetoclax e hipometilantes diagnosticados de leucemia mieloide aguda (LMA). Sangre (Barc) 41(Suplem):57–58

[5] DiNardo CD et al (2020) 10-day decitabine with venetoclax for newly diagnosed intensive chemotherapy ineligible, and relapsed or refractory acute myeloid leukaemia: a single-centre, phase 2 trial. Lancet Haematol 7:e724–e736 32896301 10.1016/S2352-3026(20)30210-6 PMC7549397

[6] DiNardo CD, Wei AH (2020) How I treat acute myeloid leukemia in the era of new drugs. Blood 135:85–96 31765470 10.1182/blood.2019001239

[7] Wei A et al (2017) Phase 1/2 study of venetoclax with low-dose cytarabine in treatment-naive, elderly patients with acute myeloid leukemia unfit for intensive chemotherapy: 1-year outcomes. Blood 130:890

[8] Wei AH et al (2019) Venetoclax combined with low-dose cytarabine for previously untreated patients with acute myeloid leukemia: results from a phase Ib/II study. J Clin Oncol Off J Am Soc Clin Oncol 37:1277–1284 10.1200/JCO.18.01600 PMC6524989 30892988

[9] Masarova L et al (2021) Single-center experience with venetoclax combinations in patients with newly diagnosed and relapsed AML evolving from MPNs. Blood Adv 5:2156–2164 33885751 10.1182/bloodadvances.2020003934 PMC8095138

[10] Jin H et al (2023) Venetoclax combined with azacitidine and homoharringtonine in relapsed/refractory AML: a multicenter, phase 2 trial. J Hematol Oncol 16:42 37120593 10.1186/s13045-023-01437-1 PMC10149010

[11] Short NJ et al (2024) Azacitidine, venetoclax, and gilteritinib in newly diagnosed and relapsed or refractory FLT3-Mutated AML. J Clin Oncol 42:1499–1508 38277619 10.1200/JCO.23.01911 PMC11095865

[12] Lachowiez CA et al (2023) A phase Ib/II study of ivosidenib with venetoclax ± azacitidine in IDH1-Mutated myeloid malignancies. Blood Cancer Discov 4:276–293 37102976 10.1158/2643-3230.BCD-22-0205 PMC10320628

[13] Daver N et al (2022) Venetoclax plus gilteritinib for FLT3-Mutated relapsed/refractory acute myeloid leukemia. J Clin Oncol 40:4048–4059 35849791 10.1200/JCO.22.00602 PMC9746764

[14] Janssen M et al (2022) Venetoclax synergizes with gilteritinib in FLT3 wild-type high-risk acute myeloid leukemia by suppressing MCL-1. Blood 140:2594–2610 35857899 10.1182/blood.2021014241

[15] Chua CC et al (2020) Chemotherapy and venetoclax in elderly acute myeloid leukemia trial (CAVEAT): a phase ib dose-escalation study of venetoclax combined with modified intensive chemotherapy. J Clin Oncol 38:3506–3517 32687450 10.1200/JCO.20.00572

[16] DiNardo CD et al (2022) Venetoclax combined with FLAG-IDA induction and consolidation in newly diagnosed acute myeloid leukemia. Am J Hematol 97:1035–1043 35583199 10.1002/ajh.26601 PMC11812953

[17] Kadia TM et al (2021) Venetoclax plus intensive chemotherapy with cladribine, idarubicin, and cytarabine in patients with newly diagnosed acute myeloid leukaemia or high-risk myelodysplastic syndrome: a cohort from a single-centre, single-arm, phase 2 trial. Lancet Haematol 8:e552–e561 34329576 10.1016/S2352-3026(21)00192-7 PMC8884174

[18] Bewersdorf JP et al (2021) Venetoclax-based combinations in AML and high-risk MDS prior to and following allogeneic hematopoietic cell transplant. Leuk Lymphoma 62:3394–3401 34477024 10.1080/10428194.2021.1966788 PMC9012492

[19] Pollyea DA et al (2022) Venetoclax and azacitidine followed by allogeneic transplant results in excellent outcomes and may improve outcomes versus maintenance therapy among newly diagnosed AML patients older than 60. Bone Marrow Transplant 57:160–166 34645926 10.1038/s41409-021-01476-7

[20] Ivanov V et al (2022) Design of the VIALE-M phase III trial of venetoclax and oral azacitidine maintenance therapy in acute myeloid leukemia. Futur Oncol 18:2879–2889 10.2217/fon-2022-0450 35852098

[21] Zhao P et al (2022) Venetoclax plus azacitidine and donor lymphocyte infusion in treating acute myeloid leukemia patients who relapse after allogeneic hematopoietic stem cell transplantation. Ann Hematol 101:119–130 34568973 10.1007/s00277-021-04674-x PMC8720738

[22] Pratz KW et al (2024) Long-term follow-up of VIALE-A: Venetoclax and azacitidine in chemotherapy-ineligible untreated acute myeloid leukemia. Am J Hematol 99:615–624 38343151 10.1002/ajh.27246

[23] Arber D et al (2022) International consensus classification of myeloid neoplasms and acute leukemia: integrating morphological, clinical, and genomic data. Blood 140(11):1200–1228 35767897 10.1182/blood.2022015850 PMC9479031

[24] Khoury JD et al (2022) The 5th edition of the world health organization classification of haematolymphoid tumours: myeloid and histiocytic/dendritic neoplasms. Leukemia. 10.1038/s41375-022-01613-1 10.1038/s41375-022-01613-1 PMC9252913 35732831

[25] Döhner H et al (2022) Diagnosis and management of AML in adults: 2022 recommendations from an international expert panel on behalf of the ELN. Blood 140(12):1345–1377 35797463 10.1182/blood.2022016867

[26] Cheson BD et al (2003) Revised recommendations of the international working group for diagnosis, standardization of response criteria, treatment outcomes, and reporting standards for therapeutic trials in acute myeloid leukemia. J Clin Oncol 21:4642–4649 14673054 10.1200/JCO.2003.04.036

[27] Heuser M et al (2021) 2021 Update on MRD in acute myeloid leukemia: a consensus document from the European LeukemiaNet MRD Working Party. Blood 138:2753–2767 34724563 10.1182/blood.2021013626 PMC8718623

[28] Oken MM et al (1982) Toxicity and response criteria of the Eastern Cooperative Oncology Group. Am J Clin Oncol 5:649–655 7165009

[29] Pollyea DA et al (2020) Venetoclax with azacitidine or decitabine in patients with newly diagnosed acute myeloid leukemia: Long term follow-up from a phase 1b study. Am J Hematol. 10.1002/ajh.26039 33119898 10.1002/ajh.26039

[30] Pratz KW et al (2022) Long-term follow-up of the phase 3 viale-a clinical trial of venetoclax plus azacitidine for patients with untreated acute myeloid leukemia ineligible for intensive chemotherapy. Blood 140:529–531 34739075 10.1093/jjco/hyab170 PMC9242001

[31] Döhner H et al (2022) ELN risk stratification is not predictive of outcomes for treatment-naïve patients with acute myeloid leukemia treated with venetoclax and azacitidine. Blood 140:1441–1444

[32] Pollyea DA et al (2022) Impact of venetoclax and azacitidine in treatment-naïve patients with acute myeloid leukemia and IDH1/2 mutations. Clin Cancer Res 28:2753–2761 35046058 10.1158/1078-0432.CCR-21-3467 PMC9365354

[33] DiNardo CD et al (2020) Molecular patterns of response and treatment failure after frontline venetoclax combinations in older patients with AML. Blood 135:791–803 31932844 10.1182/blood.2019003988 PMC7068032

[34] Stahl M et al (2021) Clinical and molecular predictors of response and survival following venetoclax therapy in relapsed/refractory AML. Blood Adv 5:1552–1564 33687434 10.1182/bloodadvances.2020003734 PMC7948282

[35] DiNardo CD et al (2019) Venetoclax combined with decitabine or azacitidine in treatment-naive, elderly patients with acute myeloid leukemia. Blood 133:7–17 30361262 10.1182/blood-2018-08-868752 PMC6318429

[36] Piccini M et al (2021) Venetoclax-based regimens for relapsed/refractory acute myeloid leukemia in a real-life setting: a retrospective single-center experience. J Clin Med 10:1684 33919958 10.3390/jcm10081684 PMC8070927

[37] Morsia E et al (2020) Venetoclax and hypomethylating agents in acute myeloid leukemia: Mayo Clinic series on 86 patients. Am J Hematol 95:1511–1521 32833294 10.1002/ajh.25978

[38] Winters AC et al (2019) Real-world experience of venetoclax with azacitidine for untreated patients with acute myeloid leukemia. Blood Adv 3:2911–2919 31648312 10.1182/bloodadvances.2019000243 PMC6849960

[39] Matthews AH et al (2022) Real-world effectiveness of CPX-351 vs venetoclax and azacitidine in acute myeloid leukemia. Blood Adv 6(13):3997–4005 35507945 10.1182/bloodadvances.2022007265 PMC9278286

[40] Labrador J et al (2022) Use of venetoclax in patients with relapsed or refractory acute myeloid leukemia: the PETHEMA registry experience. Cancers 14(7):1734 35406512 10.3390/cancers14071734 PMC8997036

[41] Gaut D et al (2020) Venetoclax combination therapy in relapsed/refractory acute myeloid leukemia: A single institution experience. Leuk Res 90:106314 32035355 10.1016/j.leukres.2020.106314

[42] Eisenmann ED et al (2022) Interaction of antifungal drugs with CYP3A- and OATP1B-mediated Venetoclax elimination. Pharmaceutics 14(4):694 35456528 10.3390/pharmaceutics14040694 PMC9025810

[43] Aldoss I et al (2019) Invasive fungal infections in acute myeloid leukemia treated with venetoclax and hypomethylating agents. Blood Adv 3:4043–4049 31816059 10.1182/bloodadvances.2019000930 PMC6963254

[44] Agarwal SK et al (2017) Management of venetoclax-posaconazole interaction in acute myeloid leukemia patients: evaluation of dose adjustments. Clin Ther 39:359–367 28161120 10.1016/j.clinthera.2017.01.003

[45] DiNardo CD et al (2018) Safety and preliminary efficacy of venetoclax with decitabine or azacitidine in elderly patients with previously untreated acute myeloid leukaemia: a non-randomised, open-label, phase 1b study. Lancet Oncol 19:216–228 29339097 10.1016/S1470-2045(18)30010-X

[46] Jonas BA, Pollyea DA (2019) How we use venetoclax with hypomethylating agents for the treatment of newly diagnosed patients with acute myeloid leukemia. Leukemia 33:2795–2804 31628431 10.1038/s41375-019-0612-8

